# Supplementary material for: Are carbon emissions trading and green financial instruments synergistic? -Comprehensive quantitative research based on content analysis
Source: PLoS One. 2024 Mar 7;19(3):e0298601. doi: 10.1371/journal.pone.0298601 (PMC10919601; doi:10.1371/journal.pone.0298601)
Supplement: S1 Data — (ZIP) [file pone.0298601.s001.zip › Data/地级市Z_F工作报告.zip!/║╙─╧/σo╤⌠╩╨/└┤╫╘╣1⁄2╓┌║┼í╛┬φ┐╦╩2╛▌═°í┐_2007.docx]

各位代表：

现在，我代表市人民政府，向大会作政府工作报告，请予审议，并请各位政协委员提出意见。

一、2006年工作回顾

2006年，市人民政府在省委、省政府和市委的正确领导下，深入贯彻落实科学发展观，积极构建和谐社会，强力推进以工兴市战略、科教兴濮战略、人才强市战略、开放带动战略、可持续发展战略和品牌城市战略，在经济建设、政治建设、文化建设和社会建设等方面都取得了新成绩，圆满完成了市五届人大三次会议确定的各项任务，实现了“十一五”发展的良好开局，连续三年被省政府授予目标管理优秀单位称号。

—— 国民经济平稳较快发展，综合实力稳步提升。初步核算，全市生产总值达到455.7亿元，比上年增长15.8%，增幅居全省第四位，分别比全国、全省平均水平高5.1和1.7个百分点。其中，第一产业增长8.3%，第二产业增长18.8%，第三产业增长12.8%。人均生产总值达到1.26万元，增长14%。

——工业经济实力进一步壮大，质量和效益明显提高。全市规模以上工业企业完成增加值221亿元，增长22.8%。其中，地方规模以上工业企业完成增加值102.8亿元，增长34.9%。规模以上工业企业综合效益指数达到268%，比上年提高了52个百分点；实现销售收入692亿元，增长29%；实现利税131亿元，增长80.3%；盈亏相抵后实现利润83亿元，增长67.2%。八大优势产业完成增加值205.9亿元，增长22.7%，占全市规模以上工业企业增加值的93.3%。年初确定的128个投资超千万元的重点工业项目，完成投资113.6亿元，为年计划的157.2%。有110个项目竣工投产，年可新增产值能力87.5亿元、利税能力10.05亿元，创造就业岗位28768个。

——社会主义新农村建设扎实推进，农村经济全面发展。粮食生产创历史新高。全市粮食总产达到225.8万吨，比上年增长11.6%。农业结构不断优化。全市优质专用小麦达到200万亩、优质水稻达到67万亩、优质专用玉米达到90万亩，分别占播种面积的65%、100%和76%。经济作物面积达到212万亩，建成了尖椒、食用菌、冬枣、鲜切花等一批特色农业基地。农业产业化经营水平进一步提高。全市农业产业化龙头企业达到387家，其中年销售额超亿元的达到11家。畜牧养殖业快速发展。全年肉、蛋、奶产量达到23万吨、29.5万吨和3.3万吨，分别增长8.1%、4.9%和31.2%。农业和农村基础设施建设得到加强。筹措2亿元新建和改建农村公路811.4公里，其中“村村通”工程完成投资1亿元，新建公路605公里，新增通油路行政村464个。新建农村沼气池30152座，超额完成省定2万座的建设任务。投资3420万元解决了农村9.5万人的安全饮水问题。大力发展劳务经济。全市培训农村劳动力10.3万人，转移输出劳务工52.5万人，收入23.5亿元。农田水利基本建设成绩显著，荣获全省“红旗渠精神杯”市级杯七连冠。林业生态建设取得新成效。完成人工造林18.3万亩，新建农田林网12万亩，完成通道绿化307公里。组织实施了“万村千乡市场工程”，共建成农家店535家。加大扶贫开发工作力度，全市共解决和巩固温饱3.3万人。第二次全国农业普查取得阶段性成果。

——新建一批基础产业和基础设施重点项目，发展后劲进一步增强。全市全社会固定资产投资完成184亿元，比上年增长30.6%。其中地方投资完成116亿元，增长42%。开工建设了170个基础产业和基础设施重点项目，完成投资137.1亿元，为年计划的140.8%，其中有132个项目竣工投产，其余项目完成了计划进度。一大批事关濮阳发展和民生的重点项目建设取得突破。金堤河治理工程又建成了台前县李皮匠沟、苗口沟和范县十字坡沟三座闸站，基本解决了范县、台前县60多万亩耕地内涝问题；备受广大市民关注的渠村引黄闸改建工程建成通水，解决了50万城市居民生活、城市工业及150多万农民的用水安全和193万亩耕地灌溉问题；国电2×20万千瓦热电机组建成并网发电；年产100万吨的同力水泥粉磨站项目竣工投产；安阳至濮阳500KV输变电工程竣工投用，县城电网改建工程全面完成；大广高速公路濮阳段建成通车，结束了我市无国家干线高速公路的历史；濮阳垃圾发电项目试车成功；濮范高速公路、龙丰纸业25万吨纸机工程、濮阳县秸秆发电等项目开工建设。中原大化年产50万吨甲醇项目完成投资15.5亿元，为年计划的194%。年初向全市人民承诺的12件实事全部落实。

——改革开放力度加大，招商引资成绩突出。国有企业改革继续深化，全市177家国有企业改制面达到97%，盘活资产1.5亿元，安置职工2300人。其中市直41户国有企业完成了改革任务，改制面达到93%。市甲醇厂20万吨甲醇扩建工程与全国100强—永煤集团正式签约。按照全省统一部署，完成了公务员工资套改。事业单位收入分配制度改革取得阶段性成效。基本完成了建设系统“三项改革”。交通和统计方法制度等改革取得新进展。招商引资成效显著。以第四届河南投洽会、世界舜裔宗亲联谊会和500多台商赴濮为平台，开展了招商活动，共签约合同117份，总投资94.7亿元。内外贸易发展迅速。全市社会消费品零售总额达115亿元，比上年增长14.7%。外贸进出口总额3.2亿美元，增长92%。其中外贸出口达到2.7亿美元，增长73%，增速居全省首位。全市新批外商投资企业21家，实际利用外资4632万美元，增长25.8%。全年新签对外承包工程和外派劳务合同额5.2亿美元，完成营业额3.8亿美元，外派人数5543人，分别占年目标的158%、147%、139%，3项指标连续8年均居全省首位。

——非公有制经济蓬勃发展，成为国民经济的“半壁江山”。全市规模以上非公有制企业达到582家。全市非公有制经济完成增加值232亿元，比上年增长21.2%；完成营业收入797.9亿元，增长16.6%；实现利润88.2亿元，增长20.5%；实交税金10.4亿元，增长20.3%。非公有制经济占全市经济总量的50.9%，比上年提高了3.8个百分点。

——财政稳定增收，金融运行平稳。全市财政一般预算收入完成18.6亿元，比上年增长21%。一般预算支出完成42.4亿元，增长21.9%。充分发挥了公共财政职能，在支持社会主义新农村建设，促进经济结构调整，完善城镇基础设施，发展社会事业，保障社会稳定等方面发挥了重要作用。金融机构各项存款余额达到341.4亿元，较年初增加26.9亿元；各项贷款余额达到177.3亿元，增加36.2亿元。各项贷款余额创2000年以来最高水平。

——城市建设和管理取得新成绩，人居环境进一步优化。以迎接国家卫生城市复检为契机，切实加强城市建设和管理，完善城市基础设施和功能，城市面貌发生新变化，国家卫生城市复检工作取得成功，被全国爱卫会重新确认为国家卫生城市。第三轮城市总体规划修编已报省政府待批，完成了历史文化名城保护等城市专项规划和5个县城的总体规划。新建、续建开州路北延、黄河路东延等城市主次干道10条，改造城市背街小巷14条，扩大了城市绿化美化面积，完善了市政、环卫等基础设施。新建了9座公厕、8座垃圾中转站。县城和小城镇建设取得新成绩。濮阳县文留镇、柳屯镇被评为“全国文明镇”。全市城镇化率达到30.32%，比上年提高1.5个百分点。在首届《中国城市生活质量报告》排行榜上，濮阳市居第125位，在省内城市中排名第5位。

——社会事业全面发展，和谐社会建设成效显著。深入实施科教兴濮和人才强市战略，组织实施各类科技项目189项，新增民营科技企业40家、省级高新技术企业5家、省级高新技术产品17种。与郑州大学、省科学院建立了市校、市院合作关系。基础教育进一步巩固，职业教育、成人教育和高等教育有了新的发展。普通高考再创佳绩，全市本科上线9318人，比上年增加1088人。落实农村中小学“两免一补”资金6339万元，符合条件的贫困学生全部发放到位。加强人口和计划生育工作，扎实开展“争先创优”活动，全市人口自然增长率达到4.71‰，低于全省平均水平。南乐县被评为“全国计划生育优质服务先进县”。大力推进文化体制改革，加快文化产业发展，丰富了群众性文化活动。文化艺术中心广场建成投用，大弦戏等4个剧种被列入国家级非物质文化遗产保护名录，濮阳县元唐兀公碑等3处遗址被批准为国家级文物保护单位。扫黄打非工作成效显著，被评为“全省扫黄打非工作先进市”。市电视编播大楼主体建成。大力实施广播电视“村村通”工程，结束了我市东部地区长期不能收看濮阳电视台无线电视节目的历史。新一代天气雷达站建成投用。广泛开展全民健身运动，竞技体育取得新成绩，在省十运会上我市代表团获得45枚金牌。进一步改善城乡居民医疗条件，改建了20所乡镇卫生院，新建了6所社区卫生服务站。台前县、濮阳县新型农村合作医疗稳步推进，参合农民84.3万人。大力发展旅游业，2006年全市接待游客231.9万人次，增长6.4%；旅游收入3亿元，增长8.9%。认真做好就业和社会保障工作，全市城镇新增就业人员5.4万人，下岗失业人员再就业1.8万人，其中“4050”人员7953人。改造和扩建了74所乡镇敬老院，农村五保户集中供养率达到省定20%的目标。巩固了“两个确保”，城区和农村居民最低生活保障标准分别提高到160元和20元，企业离退休人员基本养老金按时足额发放率和社会化发放率均为100%。我市成为全省第一个扩大失业保险基金支出范围试点市。人民生活水平继续提高，全市城镇居民人均可支配收入8963元，增长12.4%；农民人均纯收入达到2907元，增长17.6%。切实加强安全生产工作，全市各类伤亡事故和死亡人数同比分别下降38.2%和24%，连续三年被省政府评为全省安全生产工作优秀市。省定目标内的清理建设领域拖欠工程款任务基本完成。土地管理、环境保护、民族宗教、外事侨务、审计、物价、史志、人防、地震、气象、保险、邮电通信、残疾人事业、国防教育、民兵预备役等各项工作都取得了新的成绩。

——精神文明和民主法制建设进一步加强，社会大局保持稳定。社会主义荣辱观教育深入开展，精神文明创建活动不断深化。加强法制宣传教育，扎实推进依法治市，认真贯彻《全面推进依法行政实施纲要》，全面推行行政执法责任制。自觉接受人大法律监督、工作监督和政协民主监督，认真办理人大代表议案、建议和政协提案。支持各民主党派、工商联民主监督、参政议政。双拥共建工作继续推进，军政军民团结进一步加强。认真开展商业贿赂等专项治理，反腐倡廉工作取得新成效。切实加强信访工作，及时化解社会矛盾，强化社会治安综合治理，严厉打击各类违法犯罪活动，连续三年被评为“河南省平安建设先进省辖市”。

各位代表，过去一年成绩的取得，是省委、省政府和市委正确领导的结果，是全市人民共同奋斗的结果。在此，我代表市人民政府向全市广大工人、农民、知识分子、干部、人民解放军指战员、武警官兵、公安民警以及各界人士致以崇高的敬意，表示衷心的感谢！

在肯定成绩的同时，我们也要清醒地看到，当前我市经济社会发展中仍然存在一些实际困难及薄弱环节，主要是：农业虽稳步发展，但基础比较薄弱，抗灾能力不强，农民增收压力较大；工业发展势头很好，但地方自主发展能力不强的结构性矛盾没有发生根本性变化；服务业在国民经济中所占比重偏低，并且有下降趋势，分别比全国、全省平均水平低19和9个百分点。同时，经济发展受市场因素制约较大，煤电油气运有效供应不足、价格上涨，影响了企业效益；部分企业流资短缺，生产经营困难等等。对此，我们一定高度重视，并在今后的工作中认真加以解决。

二、2007年工作的总体要求、奋斗目标和主要任务

2007年是我市深入贯彻科学发展观、加快濮阳发展的关键一年，经济社会发展面临新的机遇和挑战。经济全球化趋势深入发展，科技创新成果加速应用和推广，国际产业结构调整步伐加快，高新技术产业和现代服务业加快转移，制造业及相关技术、资本加快向投资环境较好的国家和地区转移，世界经济仍将保持较快增长势头。国家加快促进中部崛起、建设社会主义新农村、构建社会主义和谐社会，继续加强和改善宏观调控，保持宏观经济政策的连续性和稳定性，将进一步促进经济社会又好又快发展。省八次党代会提出了加快“两大跨越”、推进“两大建设”、奋力实现中原崛起的历史任务，为我们指明了前进的方向。经过近几年的努力，濮阳经济持续快速发展，2004年至2006年，全市年均GDP增速达到15.1%，是历史上增长最快的时期，经济加速发展的势头非常明显，我们已经具备了加快发展的良好基础和必要条件。同时，也要看到，世界能源资源价格波动，贸易保护主义有所抬头；经济加快发展与人口、资源、环境以及增长方式粗放的矛盾日益突出，区域竞争不断加剧；我市经济社会发展与省内先进市相比还有一定差距，一些主要经济指标增幅仍处于全省落后位次。因此，当前我市面临着有利条件大于不利因素、经济发展环境相对较好的总体形势，我们要按照市五次党代会提出的建设富裕文明和谐濮阳的奋斗目标，围绕实现经济强市、文化强市两个新跨越，大力推进濮阳建设和发展。

今年政府工作的总体要求是：以邓小平理论和“三个代表”重要思想为指导，认真贯彻党的十六届六中全会、省八次党代会和市五次党代会精神，坚持以科学发展观统领经济社会发展全局，加快和谐社会建设，围绕全面建设小康社会和建设富裕文明和谐濮阳的目标，大力实施以工兴市战略、科教兴濮战略、人才强市战略、开放带动战略、可持续发展战略和品牌城市战略，强力推进工业化、城镇化和农业现代化，着力调整经济结构和转变增长方式，着力加强资源节约和环境保护，着力推进改革开放和自主创新，着力促进社会发展和解决民生问题，努力实现经济社会又好又快发展。

今年经济社会发展的预期目标是：生产总值增长13%；地方财政一般预算收入增长13%，力争达到15%；全部工业增加值增长16%；全社会固定资产投资增长18%，力争达到20%；社会消费品零售总额增长14%；外贸出口增长15%，实际利用外资增长30%；居民消费价格涨幅控制在2.5%以内；城镇居民人均可支配收入增长8%，农民人均纯收入增长8%；单位生产总值能耗降低4.4%，化学需氧量、二氧化硫排放量分别减少2.4%和1%；人口自然增长率控制在6.5‰以下；城镇登记失业率控制在4%以内，城镇新增就业人数6万人。完成上述目标，要重点抓好以下十个方面的工作：

（一）大力推进产业结构优化升级，壮大工业经济实力。要围绕资源型工业城市转型，坚定不移地实施以工兴市战略。按照“龙头带动、产业支撑、优化结构、集群发展”的思路和新型工业化的要求，更加注重工业结构调整和产业升级，更加注重企业自主创新能力的增强，全面提升工业综合实力和竞争力。一要优化工业结构，壮大优势产业。重点支持石油化工、林纸林板、特种玻璃及电光源、食品加工、冶金建材、机械设备制造、纺织、电力等八大优势产业优化结构，转变增长方式，拉长产业链条，发展精深加工产品、终端产品和高档产品，提升产品档次。政府要牵线搭桥，在政策和资金等方面支持鼓励外资和民间资本放大和延伸我市优势产业，使资源优势、产业优势变成经济优势和竞争优势。二要抓好龙头项目建设，促进产业化发展。要下大气力做好世界500强和国内500强企业的引进工作，新上一批大项目、科技含量高的项目、能形成和带动产业发展的龙头项目，提升濮阳形象，增强濮阳实力。要大力支持中原油田、中原乙烯、中原大化、濮耐、龙丰纸业、力诺玻璃、中原总机厂、全力食品、三强集团等八大优势产业中的企业龙头，发挥优势，做强做大，增强产业竞争力和带动力。要整合八大优势产业的资源，优化结构，集群发展，制定标准，培育品牌，实现新的跨越。同时，今年要集中力量抓好122个投资超千万元的重点工业项目建设，形成新的增长点。三要发展园区经济，打造产业集群。要把高新区和濮阳工业园区建成濮阳对外开放的窗口、新型产业发展的示范区和经济发展的动力基地。高新区要搞好功能区详细规划及发展规划，狠抓招商引资和基础设施建设，完善服务功能，壮大经济实力。工业园区要借鉴上海及长三角地区的先进经验，制定科学的管理体制和运作模式，走科学化、规范化发展的道路。各县（区）也要加强本级工业园区的配套完善，使新项目向工业园区聚集。

（二）大力推进社会主义新农村建设，全面发展现代农业。要认真落实中央、省建设社会主义新农村的战略部署，坚持实行工业反哺农业、城市支持农村和多予少取放活的方针，扎扎实实做好农业和农村工作，全面发展农村经济，促进农民持续增收。一要加强现代农业产业体系建设。要搞好农业综合开发，加强耕地特别是基本农田保护，抓好濮阳县、清丰县、南乐县国家优质小麦基地和范县、濮阳县优质无公害水稻生产基地建设，全市粮食产量稳定在190万吨以上，争取达到200万吨。要加强畜产品生产、加工基地建设，扩大146个标准化畜禽养殖小区的生产规模。要积极发展畜产品精深加工，实现畜产品加工增值。发展稻鸭共作、水产养殖。积极发展特色农业、品牌农业和标准化农业，培育特色主导产业，进一步扩大尖椒、花卉、食用菌等高效经济作物面积。加强农产品安全监测、无公害农产品基地认定、绿色食品和有机食品认证工作。要重视林业生态建设，大力营造速生丰产林，发展林下经济，提高林业综合效益。要大力扶持73家农业产业化重点龙头企业发展，加快构筑现代农业产业体系。要积极发展适应现代农业要求的物流产业，鼓励发展农民专业合作经济组织，健全农村社会化服务体系。二要加快农村基础设施建设和社会事业发展。切实把基础设施建设和社会事业发展的重点转向农村，财政新增教育、卫生、文化等经费和固定资产投资增量主要用于农村，逐步加大政府土地出让金用于农村的比重，着力解决农村农田水利、饮水安全、农村公路、清洁能源、农村电网和广播电视等突出问题。要加强农田水利基本建设，继续做好引黄灌区续建配套和节水改造工程建设，年内新增有效灌溉面积2万亩，新增节水灌溉面积6万亩，新增除涝面积8万亩。要加快农村供电、通讯等基础设施建设。继续实施农村电影放映“2131”工程，基本实现每村一月放映一次电影，继续推进广播电视“村村通”工程、文化信息资源共享工程。三要推动新农村建设取得实质性进展。加强对新农村建设的规划指导和扶持。深入开展“抓试点、争致富、办实事”活动，搞好306个试点村建设，完善村内道路、给排水和垃圾处理等公共设施，搞好村庄绿化，改善农村环境卫生和村容村貌。培育新型农民，推动劳务经济发展，促进农民增收、农村发展和乡风文明。

（三）大力推进基础产业和基础设施项目建设，着力办好事关民生的12件实事。要加大投资力度，新上一批符合国家产业政策的大项目，保持固定资产投资的持续增长，为经济社会发展注入新的动力。今年，要抓好基础产业和基础设施方面186个投资超千万元的项目，概算总投资187亿元，年内计划完成投资100亿元。重点抓好中原大化50万吨/年甲醇、龙丰纸业25万吨/年轻量涂布纸、市甲醇厂20万吨/年甲醇扩建，抓好濮阳县华宝玻璃5万吨/年电光源玻管、和兴食业4万只/日肉鸡分割项目，清丰县恒力佳泰1500吨/年食用菌加工、七点半食品公司15万吨/年食品饮料加工项目，南乐县鹏程6000吨/年木糖及木糖加工、宏业化工5万吨/年过碳酸钠项目，范县兴发玻璃2亿只/年圣诞饰品、龙马实业4万吨/年铜加工项目，台前县恒润石化2万吨/年高档石油树脂加工、绿源牧业500头/日肉牛分割加工，华龙区瑞杰实业3500吨/年石油助剂加工、华龙科技1万吨/年玻璃纤维加工项目，高新区颖泰化工扩建、新力化工汽车专用高档涂料等大型项目建设。抓好濮范高速公路、南林高速公路濮阳段建设，力争开工建设德商高速公路范县段、大广高速公路北延至省界段工程。做好龙丰纸业二期工程30万吨浆、50万吨纸，中原乙烯30万吨扩建以及范县、清丰县、濮阳县盐化工项目的前期工作。积极配合和服务榆林至济南天然气管道濮阳段、长泰铁路濮阳段工程等建设项目。要根据国家宏观经济政策和产业投资方向，围绕八大优势产业再论证充实一批高科技、高附加值的重大项目，争取进入国家、省大盘子。搞好重点项目建设，要坚持用市场机制的办法解决建设资金不足问题，充分发挥市场配置资源的作用，强化企业和民间资本的投资主体地位，拓宽外资、民资和社会资金的融资渠道，引导更多的资金进入基础产业和基础设施建设领域。

今年市政府要办好与全市人民群众生产生活息息相关的12件实事：（1）加大对农民的补贴力度。积极筹措资金，继续实施对种粮农民直接补贴、农业生产资料综合直补、优质专用小麦良种补贴、专用玉米良种补贴、农村劳动力转移培训补贴、测土配方施肥补贴、农机具购置补贴，确保补贴金额高于上年。（2）继续改善农民基本生产生活条件。筹措资金3500万元，改建“村村通”公路200公里，完善黄河滩区村村通水泥路，建设150个农村招呼车站，完善农村客运设施。筹措资金2880万元，解决8万人以上农村居民饮水安全问题。筹措资金8000万元，用于无电村户和农网盲点村电力建设，实现农民用上电、用好电、同价电。筹措资金2000万元，扶持农村沼气工程建设，新建2万户，在沼气推广成规模的地区建设沼气服务站。（3）巩固提高农村义务教育水平。全部免除农村义务教育阶段学杂费，继续对困难家庭学生免费提供课本并补助寄宿生生活费。从2007年起，用两年时间把贫困地区农村中小学课桌凳更新配置一遍。筹措资金2550万元，用于农村176所中小学校舍维修改造。筹措资金3125万元，用于农村930所中小学和94个教学点的现代远程教育工程建设。（4）缓解群众看病难、吃药贵问题。筹措资金2000万元，完成19所乡镇卫生院的改造任务，增加基本医疗设备配置。改造贫困县医院和妇幼保健院。在全市农村全面开展新型农村合作医疗。按上级部署对价格偏高药品进行分期分批降价。（5）加强基层组织建设。筹措资金1000万元，完成216个村级组织活动场所建设。加强基层司法所建设，积极建设平安乡村。（6）做好农村劳动力转移就业工作。继续实施农村劳动力技能就业计划、农村劳动力培训阳光工程、贫困地区劳动力转移培训雨露计划，完成引导性培训7万人、技能性培训3万人，确保全年新增5万农村富余劳动力转移就业。认真解决进城务工农民子女上学问题。（7）加强城镇就业再就业和社会保障工作。全面落实就业再就业优惠政策，积极促进下岗失业人员再就业，加大对“4050”人员、城镇低保人员等困难群体再就业的帮扶力度，确保全年城镇新增就业6万人，消除零就业家庭500户以上，实现下岗失业人员再就业1.1万人、其中“4050”人员4000人。扩大社会保险覆盖面，城镇基本养老保险参保人数达到11.8万人、城镇基本医疗保险参保人数24.8万人。（8）加大对城乡困难群体的救助力度。完善农村低保制度，适当提高农村低保补差标准，确保每人每月补差不低于30元。加强农村敬老院建设，农村五保户集中供养率提高到30%。增加对城乡医疗救助的资金补助，确保补助金额高于上年。加大扶贫开发力度，力争解决和巩固3.1万农村贫困人口温饱问题。（9）加强区域污染防治。所有县城建成污水处理项目和生活垃圾处理场，建立运营长效机制，确保已建成污水处理项目和垃圾处理场正常运行。（10）完善城市基础设施。筹措4000万元资金，完成石化路东延及10条背街小巷改造，安装黄河路、开州路等新修路段路灯，建设10座公厕、10座垃圾中转站。筹资1000万元，对颐和广场、濮水公园等城市公共绿地进行高标准改造和建设。筹资500万元，建设戚城文物景区西大门。（11）加强城区教育基础设施建设。筹措2800万元资金，建成市第九中学教学楼并投入使用，开工建设油田六中综合楼等项目。（12）加快经济适用房和廉租住房建设。争取新开工建设经济适用房5万平方米，逐步使城市低收入家庭住上经济适用房。建设廉租住房5000平方米，解决100户城市困难家庭的住房问题。

（四）大力推进现代服务业发展，增强消费对经济发展的拉动力。一要以物流业和建筑业为突破口，大力发展现代服务业。要充分利用我市交通网络比较健全、区位优势明显、八大产业发展势头强劲和中央、省属大企业已经形成物资供应和销售物流链的有利条件，抓紧制定规划，建设濮阳现代物流港。要完善设施，积极发展商贸流通业。要扩大“万村千乡市场工程”实施范围，加快农家店建设进度，推动农村现代商品流通体系建设。要进一步加大对建筑业发展的扶持力度，培育壮大骨干建筑企业，大力发展施工总承包和建筑劳务分包。要加快建筑劳务基地建设，努力增加就业，促进服务业发展提速、比重提高。支持房地产业健康发展，调整住房供应结构，加快建设廉租住房和中小套型、中低价位普通商品住房，改进和规范经济适用住房制度，构建多层次住房保障体系。同时，要大力发展金融保险、研发设计、投资与资产管理等生产性服务业，鼓励发展评估、信息、会计、法律、咨询等中介服务业，加快发展社区服务、物业管理、电子商务等新兴服务业。二要加快发展旅游业。制定旅游发展规划，全面落实加快旅游业发展的各项政策措施，加强旅游基础设施建设，完善旅游服务功能，改善旅游环境，进一步充实绿色庄园、濮上园、世锦园、气象科技馆等旅游景点的旅游项目，打造旅游品牌，提升旅游产业水平。三要改善消费环境，正确引导消费需求，不断拓宽消费领域，以消费结构升级带动产业结构调整。

（五）大力推进对外开放，全面提升外向型经济发展水平。一要实现从招商引资向招商选资转变。要从加快产业结构优化升级出发，紧紧围绕八大产业、核心企业抓招商，围绕延长产业链条、增加财政收入抓招商。要对外来资金和项目，优中选优，积极引进符合产业发展方向的高科技项目、大项目和战略投资者。要坚持科学招商，以对濮阳长远发展和对子孙后代负责的态度，坚决摒弃高耗能、多占地、高污染的项目，拒绝污染搬家。二要拓宽招商渠道，创新招商方式。要充分发挥企业招商的主体作用，加强专业招商队伍建设。要丰富园区招商、产业链招商、代理招商和以商招商等，对重大项目一事一议，全力支持项目建设和企业发展，争取更多的大项目落户濮阳。三要多渠道、多形式组织参加国家、省举办的国内外大型招商活动。市里统一组织1至2次赴经济发达地区的招商活动，要精心筹划，确保取得实效。四要加大已签约项目的落实，切实提高招商实效。要进一步改善投资环境，建立完善项目落实机制，对引进的项目要全程跟踪问效，确保在较短的时间内能够开工生产。严防出现“纸上项目”和“半拉子工程”。五要认真抓好外经外贸工作，扩大出口拉动。要优化出口商品结构，支持具有自主知识产权、科技含量高、附加值高的产品扩大出口，增强竞争力。要大力开拓外经工作，积极发展对外工程承包和劳务合作。

（六）大力推进改革进程，为科学发展提供动力和体制机制保障。一要继续深化国有企业改革。以提高国有企业竞争力为重点，加大国有企业改革力度，既要通过股份制改造、引入战略投资者等方式实现产权多元化，加快培育大型企业集团，又要通过多元化改革促进企业经营机制转换，完善法人治理结构，增强企业的活力和竞争力。要加快市三强集团、市甲醇厂等国有工业企业改革进程。年内基本完成市直单位所属企业改革。全面推进国有非工业企业改革，今年全市完成30%的改革任务。进一步健全国有资产监管体制，加强国有企业及国有资产监管，完善企业法人治理结构、投资风险控制机制，严格产权交易和股权转让程序，防止国有资产流失，确保国有资产保值增值。二要深化农村综合改革。深化乡镇机构改革，建立乡镇机构和人员编制管理的长效机制，确保乡镇机构和人员编制今后5年只减不增。以落实教育经费保障机制为重点推进农村义务教育改革，确保各级财政应承担的公用经费按规定增加、编制内教师工资按现行标准及时足额发放、农村中小学校舍维修改造资金足额落实。以增强基层财政保障能力为重点推进县乡财政管理体制改革，完善“乡财县管”制度。继续深化农村信用社改革，探索发展适合农村特点的新型金融组织。三要认真组织实施公务员工资分配制度改革，积极调整支出结构，统筹安排预算内、外财力，压缩一般性支出，确保工资和津贴补贴发放。要认真开展行政事业单位资产清查，提高资产管理水平。四要深化文化体制改革。积极搞好文化体制改革试点，对国有文化企业事业单位进行分类，加快公司制改造和经营性文化事业单位转企改制，促进文化事业单位经营业务与主业剥离，增强发展活力。五要深化重要资源性产品价格改革，加强价格监管，维护广大人民群众的合法权益。同时，扎实推进建设系统、交通领域的各项改革，深化事业单位人事制度改革、科技体制改革和科研机构改革，继续推进公共财政、粮食流通、医疗卫生等领域的改革。

要充分认识非公有制经济在促进经济增长、扩大就业、繁荣市场等方面的重要作用，进一步放宽市场准入，加大扶持力度，营造宽松环境，鼓励非公有制经济参与国有和城镇集体企业产权制度改革，参与城市基础设施和市政公用设施建设，参与农业综合开发。要拓宽投融资渠道，引导商业银行加大对非公有制企业的信贷支持力度。要重点扶持比较优势明显、带动作用强的非公有制企业，引导其走规模型、科技型、外向型的发展道路，努力实现我市非公有制经济发展新的突破。

（七）大力推进区域性中心城市建设，壮大城市经济和县域经济。要把城市建设放到经济社会发展大格局中来把握，建管并重，区域性中心城市、县城、小城镇“三头并进”，努力构建资源节约、环境友好、经济高效、社会和谐的城镇发展新格局。要发挥规划在城市建设中的龙头作用，高标准编制和完善各项城市专业规划，提高城市详细规划覆盖率，严格城市规划管理，提高城市的档次和水平。要结合迎接国家园林城市复检工作，完善提高园林绿化水平。进一步加强城市基础设施建设，加快市政道路、污雨水泵站、环卫、路灯等城建重点项目的建设，拉大城市框架，完善城市功能，增强城市承载能力。大力开展城市精细化管理活动，加快城市建设和管理向高品位、高质量、精细化转变，建立健全长效管理机制，巩固和发展“六城二奖”成果，建设宜居城市，树立濮阳新形象。要统一规划、制定方案，推进“城中村”改造工作。要加快发展县城和小城镇，推进5个县城和文留、柳屯等10个重点镇建设，努力培育一批工业强镇、商贸富镇、交通枢纽重镇。力争全市城镇化率达到32%。

要大力发展城市经济。一个地区经济发展水平的高低，在很大程度上取决于城市发展的规模、质量和持续性。要着眼长远，加快资源型城市经济结构转型，通过招商引资，吸引社会资金发展替代产业，做大城市经济规模。要大力发展劳动密集型产业，创造就业岗位，增强城市吸纳能力。

要大力发展县域经济。要鼓励综合实力较强的县放大优势、率先发展，把功夫下在高起点推进结构调整、增强自主创新能力、扩大对外开放、培植优势产业和骨干企业集群上，不断提升经济整体素质和竞争实力。要支持经济基础较为薄弱的县自加压力，知难而进，用足用好扶持政策，在夯实基础、膨胀规模、增强后劲上见到大的成效，争取综合实力排名在全省位次前移。要组织市直有关部门及时提供政策、信息、人才、项目等各方面的服务，支持县（区）发展特色经济，壮大县域经济实力。

（八）大力推进财税金融工作，提高对经济社会发展的支撑能力。要坚持把财源建设与产业发展紧密结合起来，培育各具特色的税收增长点，努力增加财力。要依法征税，尤其要加强对重点税种、重点行业和重点企业的征管，严格控制减免税收，严厉打击偷、逃、骗税行为。严格落实“收支两条线”，加强非税收入管理，增强政府调控能力。要优化财政支出结构，统筹安排好财政支出，重点用于工资制度改革、地方津贴补贴改革政策和资金的落实，用于保障重点事业发展。要把更多的资金投向公共服务领域，推进公共服务均等化，不断加大对重点支出项目的保障力度，向农村倾斜，向社会事业发展的薄弱环节倾斜，向困难基层、困难群众倾斜，不断改善人民群众的生产生活条件，让广大人民群众共享改革发展成果。要进一步加强财政管理和监督，严格预算约束，加大审计执法力度，严肃财经纪律，提高财政资金的使用效益。

要落实金融政策，突出信贷工作重点，加大信贷投放力度，加强银企合作，确保重点企业、重点建设项目的资金需求。同时，要积极调整信贷结构，扩大对有市场、有效益、有信用的中小企业、民营企业的贷款规模，继续加大对“三农”的信贷支持。要及时协调解决银企合作中出现的新情况、新问题，为金融机构创造良好的经营环境。

（九）大力推进和谐濮阳建设，认真解决民生问题。要关注民生、重视民生、保障民生、改善民生，认真解决群众最关心、最直接、最现实的利益问题，扎实推进和谐濮阳建设。一要切实做好就业再就业工作。要完善和落实小额贷款、税费减免等就业再就业扶持政策，大力发展就业吸纳能力强的劳动密集型产业、中小企业和服务业，加大公益性岗位开发力度，重点做好下岗失业人员再就业工作，重点帮助“4050”人员等困难群体就业，基本解决“零就业”家庭就业问题。加强公共就业服务体系建设，广泛开展职业培训、职业介绍和就业指导。高度重视高校毕业生就业问题。二要加快社会保障体系建设。要积极扩大城镇社会保险覆盖面，尽快使养老、医疗、失业、工伤和生育等社会保险覆盖到各类企业、事业单位和个体工商户。要抓好做实基本养老保险个人账户试点工作。要完善城市居民最低生活保障制度，实现动态管理下的应保尽保。要规范农民工劳动管理制度，积极推行适合农民工特点的工伤、医疗、养老等社会保障制度。发挥商业保险在健全社会保障体系中的重要作用。要加强对社会保险基金、住房公积金的监管，确保基金安全。三要保持社会大局稳定。要全面落实安全生产责任制，加强安全生产应急救援工作，切实抓好危险化学品、交通运输、烟花爆竹及消防安全等重点领域的专项整治，加强对公共场所和重要设施的安全管理，坚决遏制重特大事故发生。要完善社会治安防控体系，依法严厉打击各种违法犯罪活动，深入开展平安创建活动，保障人民群众安居乐业。要创新社区管理体制，加快社区建设步伐，增强社区综合服务功能。要落实信访工作责任制，高度重视并认真解决土地征用、房屋拆迁、企业改制、食品药品安全以及建设领域拖欠工程款和农民工工资等方面人民群众反映强烈的问题，及时化解各种社会矛盾。要进一步整顿和规范市场经济秩序，创造良好发展环境。四要加快建设资源节约型和环境友好型社会。要强化节能降耗工作，严格执行重点产品能耗限额标准，加强高耗能行业节能管理，淘汰落后生产能力，依法关闭破坏资源和污染环境的企业。要全面推进建筑节能工作，加快发展新型墙材，确保“禁实”目标顺利完成。要加大环境保护力度，确保城市饮用水源水质稳定达标、城市空气质量进一步提高。要完善资源有偿使用机制，推行土地集约利用，合理保护和利用自然资源，建设节约型社会，促进人与自然和谐发展。

（十）大力推进各项社会事业发展，进一步加强精神文明和民主法制建设。大力实施科教兴濮战略和人才强市战略。加强科技交流与合作，搞好科技成果的引进、示范和推广，提高科技成果转化率，重点支持有创新能力的企业发展，努力建设创新型城市。认真实施《全民科学素质行动计划纲要》。坚持教育优先发展，巩固和提高义务教育，扩大高中阶段教育规模，大力发展职业教育、成人教育，规范发展民办教育，提高高等教育质量。积极做好“两基”国家验收准备工作。支持一批县级职教中心和示范性中等职业学校建设。完善人才公共服务体系，加强创新型人才和各类紧缺人才的培养、选拔和引进，促进人才资源的合理配置。大力开展爱国卫生运动。完善城乡医疗卫生服务体系，加快建立城市社区卫生服务网络，启动以大病统筹为主的城镇居民医疗保险试点，使人民群众得到更多的实惠。深入开展“争先创优”活动，稳定低生育水平，加强基层基础工作，落实奖励扶助政策，统筹解决出生人口性别比偏高、流动人口管理、人口老龄化等问题，确保实现人口控制目标。大力发展体育事业，加强城乡体育设施建设，增强人民群众体质。围绕建设文化强市，加强基础文化设施建设，完善公共文化服务体系，发展地方特色文化产业，打造文化精品，不断满足人民群众文化需求。精心举办第五届濮阳中华龙文化节和第二届杂技艺术节，支持南乐县举办“仓颉·汉字”文化节。加强文化市场监管，深入开展“扫黄打非”斗争。加强文物的保护、开发和利用。继续做好外事侨务、统计、地震、气象、史志、邮电通信、残疾人事业等工作。

以社会主义核心价值体系为重点，加强理想信念教育和思想道德建设。大力开展爱国主义、集体主义、社会主义教育，深化道德规范进万家、诚实守信万人行活动，推动社会公德、职业道德、家庭美德建设。继续开展生理、心理、伦理教育，加强和改进未成年人思想道德建设，抓好大学生思想政治教育工作。拓展文明城市、文明景区“双创”活动，扎实推进全国文明城市创建工作，广泛开展文明行业、文明单位和文明村、文明户创建活动，组织好文化、科技、卫生“三下乡”和科教、文体、法律、卫生“四进社区”活动，提高城乡文明程度。坚持依法治市。深入开展普法教育，提高全民法律素质。认真落实监督法，自觉接受人大及其常委会的法律监督、工作监督和人民政协的民主监督，严格执行人大及其常委会的决议、决定，支持人大代表依法行使职权，认真办理人大代表议案、建议和政协提案。广泛听取各民主党派、工商联的意见和建议，充分发挥工会、共青团、妇联等人民团体的桥梁纽带作用。全面贯彻党的民族宗教政策，积极做好民族宗教工作。加强法律援助，依法保障公民特别是妇女、未成年人和残疾人的合法权益。大力发展以扶老、助残、救孤、济困为重点的社会福利和慈善事业。

积极支持部队和国防建设。深入开展国防教育，健全国防动员体系。关心和支持驻濮解放军和武警部队建设。抓好人民防空和民兵预备役工作。认真落实军人优待抚恤和退伍安置政策，做好军队转业干部、退役军人安置工作。深入开展双拥模范城创建活动，巩固和发展军政军民团结。

三、加强政府自身改革和建设

全面落实科学发展观，努力构建社会主义和谐社会，实现经济社会又好又快发展，对政府行政能力提出了更高的要求。要着力推进政府自身改革和建设，为加快濮阳发展提供有力保障。

一是加快服务型政府建设。深化行政管理体制改革，加快职能转变，积极为各类市场主体提供优质服务、创造良好发展环境。在搞好经济调节和市场监管的同时，切实加强社会管理和公共服务职能，维护社会公平与正义。加强应急管理体系建设，有效应对自然灾害、事故灾害、公共卫生和社会安全事件，提高危机管理和抗风险能力。大力推进政务公开，确保权力公开透明运行。全面推行学校、医院、供水、供电等公用事业单位办事公开制度，保障人民群众的知情权、参与权和监督权。完善重大事项集体决策、专家咨询、社会公示与听证、决策评估等制度，推进决策的科学化、民主化。加快电子政务建设。进一步减少和规范行政许可，简化办事程序，完善限时办结、首问负责、服务承诺等制度，切实办好行政服务大厅，更好地为基层、企业和公众服务。

二是全面推进依法行政。切实加强政府法制工作，强化行政执法监督，严格实行行政执法责任制和执法过错责任追究制，搞好行政复议，依法规范行政权力运行。加强审计监督、效能监察，做到有权必有责、用权受监督、违法要追究。建立健全符合科学发展观要求的经济社会发展综合评价体系和考核指标体系，开展政府绩效评估，建立行政问责制度。在土地征用、房屋拆迁、企业改制、环境整治等方面，严格按法定程序办事，坚决纠正损害群众利益的行为。要按照经济规律和国际惯例的要求，打造法治化、制度化、规范化的服务环境。要全面落实促进经济发展的各项政策，创造诚信法治、公平竞争的市场环境，依法保护投资者的合法权益。

三是切实加强政风建设。认真学习胡锦涛总书记在中纪委第七次全会重要讲话精神，深入开展“讲正气、树新风”教育活动。弘扬求真务实精神，大兴求真务实之风，多做事关全局、谋划长远、夯实基础的工作，多解决影响改革发展稳定的突出问题，多办为人民群众排忧解难的实事。要大力精简会议、文件，切实改进会风、文风，进一步清理评比达标表彰活动，力戒形式主义和官僚主义。树立终身学习思想，不断更新知识，提高理论素养和业务能力，建设学习型机关。加强和完善目标管理责任制，狠抓各项工作的落实，建设责任政府。认真落实《公务员法》，加强公务员管理，努力建设一支政治坚定、业务精通、清正廉洁、作风优良的公务员队伍。

四是认真搞好廉政建设。要按照中纪委第七次全会和国务院第五次廉政工作会议的部署和要求，围绕规范行政权力运行，推进体制改革和制度建设，继续抓好商业贿赂专项治理和解决损害群众利益的突出问题。牢记“两个务必”，坚决抵制拜金主义、享乐主义和奢靡之风。加强节约型机关建设，坚持勤俭节约，严格财政支出管理，严格控制修建楼堂馆所，严格公费出国出境管理，严格规范公务接待行为。加强机关自身建设，继续深入实施“治庸计划”，推行效能问责制，抓好政府工作人员的教育、监督和廉洁自律，加强对重点领域的监管，严肃查处各类违纪违法案件，把政府廉政建设和反腐败斗争工作引向深入。 各位代表！做好今年的政府工作，意义重大。让我们在省委、省政府和市委的领导下，高举邓小平理论和“三个代表”重要思想伟大旗帜，全面贯彻落实科学发展观，积极推进社会主义和谐社会建设，上下一心，奋发图强，努力开创我市经济社会发展新局面，以优异成绩迎接党的十七大胜利召开！
